# Anti-IL-6 eluting immunomodulatory biomaterials prolong skin allograft survival

**DOI:** 10.1038/s41598-019-42349-w

**Published:** 2019-04-25

**Authors:** Mayuko Uehara, Xiaofei Li, Amir Sheikhi, Nooshin Zandi, Brian Walker, Bahram Saleh, Naima Banouni, Liwei Jiang, Farideh Ordikhani, Li Dai, Merve Yonar, Ishaan Vohra, Vivek Kasinath, Dennis P. Orgill, Ali Khademhosseini, Nasim Annabi, Reza Abdi

**Affiliations:** 1 Transplantation Research Center, Renal Division, Brigham and Women’s Hospital, Harvard Medical School, Boston, MA 02115 USA; 2 Biomaterials Innovation Research Center, Division of Biomedical Engineering, Department of Medicine, Brigham and Women’s Hospital, Harvard Medical School, Cambridge, MA 02139 USA; 3 Harvard-MIT Division of Health Sciences and Technology, Massachusetts Institute of Technology, Cambridge, MA 02139 USA; 4 Department of Bioengineering, University of California, Los Angeles, CA 90095 USA; 5 Center for Minimally Invasive Therapeutics (C-MIT), California NanoSystems Institute (CNSI), University of California, Los Angeles, CA 90095 USA; 6 Institute for Nanoscience and Nanotechnology, Sharif University of Technology, Tehran, Iran; 7 Department of Chemical Engineering, Northeastern University, Boston, MA 02115 USA; 8 Division of Plastic Surgery, Brigham and Women’s Hospital, Harvard Medical School, Boston, MA 02115 USA; 9 Department of Chemical and Biomolecular Engineering, University of California Los Angeles, Los Angeles, California 90095 USA; 10 Department of Radiological Sciences, David Geffen School of Medicine, University of California, Los Angeles, CA 90095 USA

**Keywords:** Immunology, Transplant immunology

## Abstract

A primary goal in the management of burn wounds is early wound closure. The use of skin allografts represents a lifesaving strategy for severe burn patients, but their ultimate rejection limits their potential efficacy and utility. IL-6 is a major pleiotropic cytokine which critically links innate and adaptive immune responses. Here, we devised anti-IL-6 receptor eluting gelatin methacryloyl (GelMA) biomaterials (GelMA/anti-IL-6), which were implanted at the interface between the wound beds and skin allografts. Our visible light crosslinked GelMA/anti-IL-6 immunomodulatory biomaterial (IMB) demonstrated a stable kinetic release profile of anti-IL-6. In addition, the incorporation of anti-IL-6 within the GelMA hydrogel had no effect on the mechanical properties of the hydrogels. Using a highly stringent skin transplant model, the GelMA/anti-IL-6 IMB almost doubled the survival of skin allografts. The use of GelMA/anti-IL-6 IMB was far superior to systemic anti-IL-6 receptor treatment in prolonging skin allograft survival. As compared to the untreated control group, skin from the GelMA/anti-IL-6 IMB group contained significantly fewer alloreactive T cells and macrophages. Interestingly, the environmental milieu of the draining lymph nodes (DLNs) of the mice implanted with the GelMA/anti-IL-6 IMB was also considerably less pro-inflammatory. The percentage of CD4^+^ IFNγ^+^ cells was much lower in the DLNs of the GelMA/anti-IL-6 IMB group in comparison to the GelMA group. These data highlight the importance of localized immune delivery in prolonging skin allograft survival and its potential utility in treating patients with severe burns.

## Introduction

Despite significant progress in the overall management of severe burns, these patients still suffer from a high mortality rate. One of the primary goals in the management of burn patients is early wound closure. In an ideal setting, permanent closure of the wound with a skin auto-graft represents the gold-standard treatment. However, implantation of skin auto-grafts is not possible in severely burned patients with massively infected wounds, donor site skin insufficiency, donor site morbidity, and lack of tolerability for additional surgery. A primary therapeutic focus in these patients is to perform early debridement and cover the wound with allografts or synthetic materials, even temporarily, to decrease the rate of infection as well as to control metabolic and fluid instability^1–5^. Transplantation of skin allografts, including cryopreserved tissues, could represent an effective strategy for wound closure in severe burn patients with insufficient skin autografts^6–11^. In the short term, skin allograft transplantation could represent a more effective strategy for wound closure, as allografts adhere more effectively and last longer than other types of synthetic substitutes. The benefits of skin allografts become even more evident with time, as these grafts become vascularized within a few days, a critically important step in providing oxygen and nutrients for the proper healing of the wound^12^. However, rejection of allografts severely limits their potential efficacy and utility. Immunosuppressive therapy in these patients is not advised, due to a marked increase in the risk of infections in burn patients. Hence, a localized immune delivery strategy that would enable long-term transplant acceptance without the need for systemic administration of immunosuppression is highly desirable in this group of patients.

A growing body of evidence highlights the importance of novel strategies that can reduce intra-graft inflammatory responses effectively^13,14^. Intra-graft inflammatory responses have increasingly received attention as an instigator of the alloimmune response^15–19^. An increase in immunostimulatory cytokines results in a pro-inflammatory milieu that tips the balance in favor of effector immune responses, as opposed to regulatory immune responses^20,21^. A key inflammatory cytokine that is recognized increasingly for its role in linking innate inflammatory immune activity to the augmentation of alloimmune responses is IL-6. IL-6 is a major pleiotropic inflammatory cytokine increases markedly in the circulation and at the site of the wound^22–24^. Higher IL-6 levels have also been associated with poorer outcomes in burn patients^25–27^.

We and others have shown that ischemic dendritic cells (DCs) produce a large amount of IL-6, a key inflammatory cytokine that downregulates regulatory T cells (Tregs) while potentiating alloreactive CD4^+^ T cells^28–32^. Altogether, accumulating experimental data now highlight the importance of innovative strategies that target intra-graft inflammation and innate immunity to improve transplant outcomes^33^.

Hydrogels are three-dimensional (3D) networks of hydrophilic polymer chains, which are crosslinked to form matrices^34,35^. They have widespread biomedical applications, due to their particular features, including tunable physical, chemical, and biological properties, biocompatibility, fabrication diversity, and resemblance to the native extracellular matrix (ECM)^36–38^. Our group and others have made significant progress in the synthesis and fabrication of hydrogels from both naturally derived and synthetic-based polymers for various applications, including regenerative medicine, drug/gene delivery, stem cell and cancer research, and cell therapy^35,36,39–44^. Engineered hydrogels that provide the controlled release of anti-inflammatory molecules have vast applications for a number of immune mediated diseases^45,46^. Another advantage of these biomaterials is their ability to preserve the activity of the incorporated compounds^47^. Nonetheless, the use of immunomodulatory hydrogels for prolonging transplant survival has been inadequately explored to date.

Here, we use a photocrosslinkable, naturally-derived hydrogel for the controlled release of anti-IL-6 to create an immune privileged bedding for skin allografts. This strategy not only has the potential to target allograft-resident effector T cells responsible for transplant rejection, but also aims to diminish intra-graft inflammatory reactions that potently instigate the alloimmune response.

## Results

### Synthesis of drug-eluting tissue adhesive immunomodulatory biomaterial (IMB)

**Figure 1:**
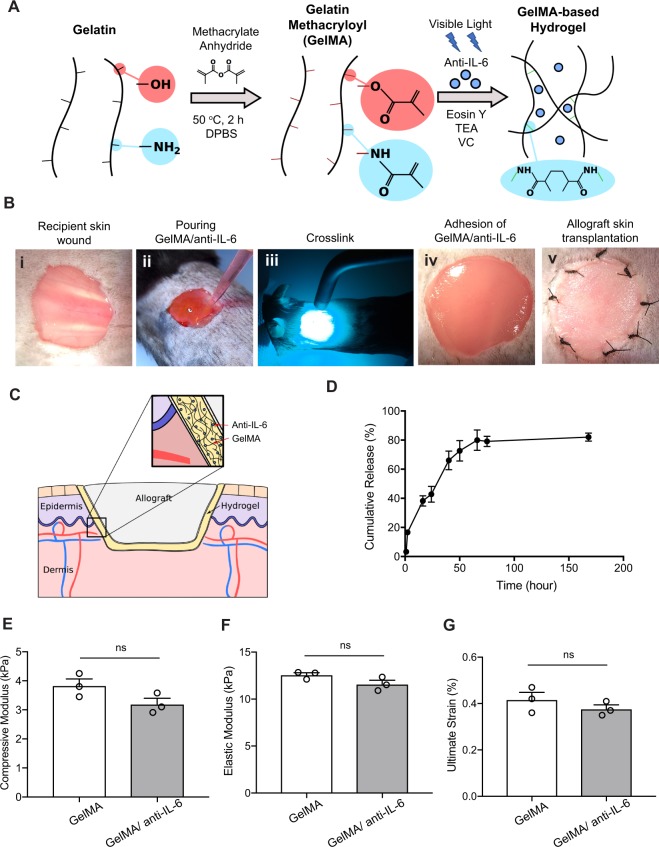
Synthesis of GelMA/anti-IL-6 immunomodulatory biomaterial (IMB). (**A**) Type A porcine skin gelatin was dissolved in Dulbecco’s phosphate buffered saline (DPBS), and methacrylic anhydride (MA, 94%) was added to the gelatin solution dropwise while stirring at 50 °C. Lyophilized GelMA was dissolved in DPBS containing TEA, VC, and anti-IL-6. The GelMA/TEA/VC/anti-IL-6 solution was mixed with the Eosin Y solution to make GelMA/anti-IL6 IMB. (**B**) A skin wound was created (i), GelMA/anti-IL-6 pre-gel solution was poured on the wound (ii), followed by exposure to the blue-green visible light (iii), yielding a tissue adhesive crosslinked hydrogel (iv), and the skin allograft was sutured onto the GelMA/anti-IL-6 hydrogel in a recipient mouse (v). (**C**) Diagram of hydrogel implantation underneath the allograft skin. (**D)** Anti-IL-6 was released gradually from GelMA/anti-IL-6 IMB. (**E)** The compressive modulus was not significantly different between GelMA control (3.8 ± 0.2 kPa) and GelMA/anti-IL-6 (3.2 ± 0.2 kPa). (*p* = 0.11, n = 3/group). (**F)** The elastic modulus was not significantly different between GelMA control (12.6 ± 0.2 kPa) and GelMA/anti-IL-6 (11.6 ± 0.4 kPa). (*p* = 0.11, n = 3/group). (**G**) Ultimate strain was not significantly different between the two groups (0.4 ± 0.03% versus 0.4 ± 0.01%). (*p* = 0.33, n = 3/group).

We synthesized an immunomodulatory biomaterial (IMB), which is a photocrosslinkable GelMA hydrogel that contains anti-IL-6 receptor (referred to as  GelMA/anti-IL-6) and can be crosslinked *in vivo* using visible light (Fig. 1A). We tested different concentrations of GelMA hydrogels for various *in vitro* and *in vivo* studies in our previous work^35^ and we found that lower concentration of GelMA (e.g. 5%) can enhance tissue ingrowth and vascularization as compared to hydrogels formed by using higher concentration of GelMA (10 or 15%). Therefore, based on these studies, we decided to use 5% GelMA in our current work. As our goal was to use GelMA/anti-IL-6 IMB in a mouse skin transplant model, the adhesion between the hydrogel and the wound was important for the success of GelMA/anti-IL-6 implantation. In our previous work, we showed that GelMA-based hydrogels have strong adhesion to the tissue and can be used as surgical adhesives and glues^37,48^. We crosslinked GelMA/anti-IL-6 *in vivo* on the skin of the mouse wound using a skin transplant model. After making a wound in the recipient mouse (C57BL/6) (Fig. 1B-i), GelMA/anti-IL-6 IMB was poured on the wound (Fig. 1B-ii) and illuminated with a blue-green visible light to form the adhesive hydrogel (Fig. 1B-iii). GelMA was successfully crosslinked and adhered to surrounding recipient skin (Fig. 1B-iv). Allograft skin (from BALB/c) was then sutured onto the GelMA/anti-IL-6 adhesive layer (Fig. 1B-v and C). The release kinetics of anti-IL-6 from GelMA/anti-IL-6 IMB showed a gradual release of anti-IL-6 over 70 hours *in vitro* (Fig. 1D).

We also characterized the mechanical properties of the engineered GelMA/anti-IL-6 IMB in comparison to pure GelMA hydrogel to confirm that the addition of anti-IL-6 to the hydrogel does not alter its mechanical stiffness. Compression and tensile tests were conducted on pristine GelMA hydrogel as well as GelMA/anti-IL-6 IMB, revealing no significant difference in compressive modulus between GelMA control (3.8 ± 0.2 kPa) and GelMA/anti-IL-6 IMB (3.2 ± 0.2 kPa) (Fig. 1E). In addition, no significant difference was found in the elastic modulus of GelMA control (12.6 ± 0.2 kPa) and GelMA/anti-IL-6 IMB (11.6 ± 0.4 kPa) (Fig. 1F). The incorporation of anti-IL-6 did not alter the value for ultimate strain (0.41 ± 0.03% for GelMA hydrogel versus 0.37 ± 0.01% for GelMA/anti-IL-6 IMB) (Fig. 1G). Taken together, these results revealed that the mechanical properties of GelMA hydrogels were not altered significantly by the addition of anti-IL-6.

### Local release of anti-IL-6 from the IMB prolongs skin allograft survival

**Figure 2:**
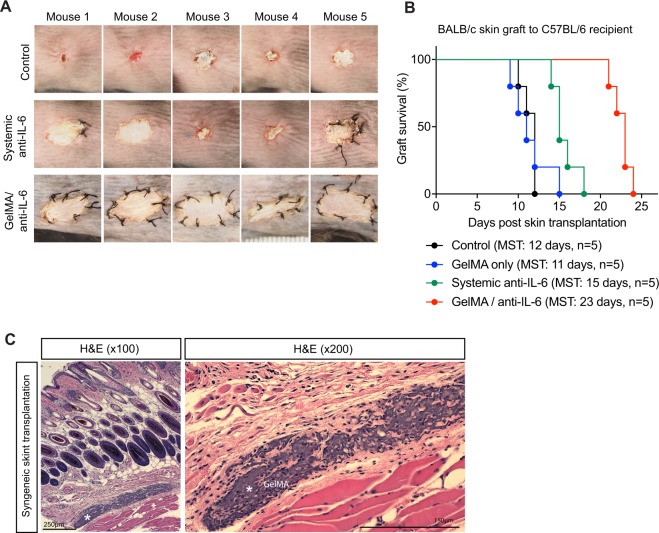
Local release of anti-IL-6 prolongs the survival of skin allograft. **(A)** Skin allografts from BALB/c donor mice were transplanted into C57BL/6 recipient mice. The skin allografts in the control group and systemic anti-IL-6-treated (100 μg/mouse iv from days 0–3 and every other day until day 11) group shrank massively and were rejected within 12 days following skin transplantation. However, the skin allografts in the GelMA/anti-IL-6 (100 μg/GelMA) group appeared intact and better tolerated. (**B)** The allograft skin survival in GelMA/anti-IL-6 group was almost double the allograft survival of control group (MST: 23 days versus 12 days, n = 5 mice/group) and significantly prolonged compared to systemic treatment with anti-IL-6 (MST: 15 days, n = 5 mice/group). GelMA implantation did not affect the allograft survival (MST: 11 days, n = 5 mice/group). (**C)** Implanted GelMA underneath the syngeneic skin graft (C57BL/6 skin graft to C57BL/6 recipient mouse) was harvested at 40 days. The remaining GelMA was attached to the surrounding tissue and degraded.

Skin from BALB/c donor mice was transplanted into fully MHC-mismatched C57BL/6 recipient mice, overlying a GelMA/anti-IL-6 adhesive layer, as demonstrated in Fig. 1B. The appearance of these skin allografts was compared with a control group, which consisted of mice that received intravenous injections of anti-IL-6 (100 μg/mouse, daily from days 0–3 and every other day until day 11; total of 800 μg of anti-IL-6), and a GelMA/anti-IL-6 (100 μg/GelMA)-implanted group (n = 5 mice/group). Twelve days post-skin transplantation, most of the skin allografts in the control group shrank markedly and were rejected (Fig. 2A, upper). However, the skin allografts transplanted with GelMA/anti-IL-6 were intact and remained attached to the wound area (Fig. 2A, lower). Surprisingly, the skin allografts in the GelMA/anti-IL-6 groups appeared healthier than the transplants in the mice treated systemically with anti-IL-6 (Fig. 2A, middle), where mice received a total amount of anti-IL-6 that was ~8-fold higher than the dose received by the GelMA/anti-IL-6 group. The implantation of GelMA/anti-IL-6 underneath the allograft skin at the time of skin transplantation nearly doubled the allograft survival, as compared to the control group (MST: 23 days versus 12 days, ***p < 0.001, n = 5 mice/group) (Fig. 2B). To confirm the biocompatibility and biodegradation of GelMA *in vivo*, GelMA was implanted underneath the syngeneic skin graft (C57BL/6 skin graft to C57BL/6 recipient mouse). Forty days after syngeneic skin transplantation, we observed that the remaining parts of GelMA were attached to the surrounding tissue (Fig. 2C). GelMA implantation had no effect on allograft survival, which was comparable to the control group in allogeneic skin transplantation (MST: 11 days versus 12 days, *p* = ns, n = 5 mice/group) (Fig. 2B). Notably, the local release of anti-IL-6 from the adhesive hydrogel exerted more profound immunoregulation with respect to prolonging skin allograft survival than systemic treatment with anti-IL-6 (MST: 23 days versus 15 days, **p < 0.01, n = 5 mice/group) (Fig. 2B). This difference indicates the potential importance of a localized immune privileged environment, which may enable a reduction in the requirement of systemic immunosuppression.

### GelMA/anti-IL-6 IMB reduces infiltration of T cells and monocytes into the skin allograft

**Figure 3:**
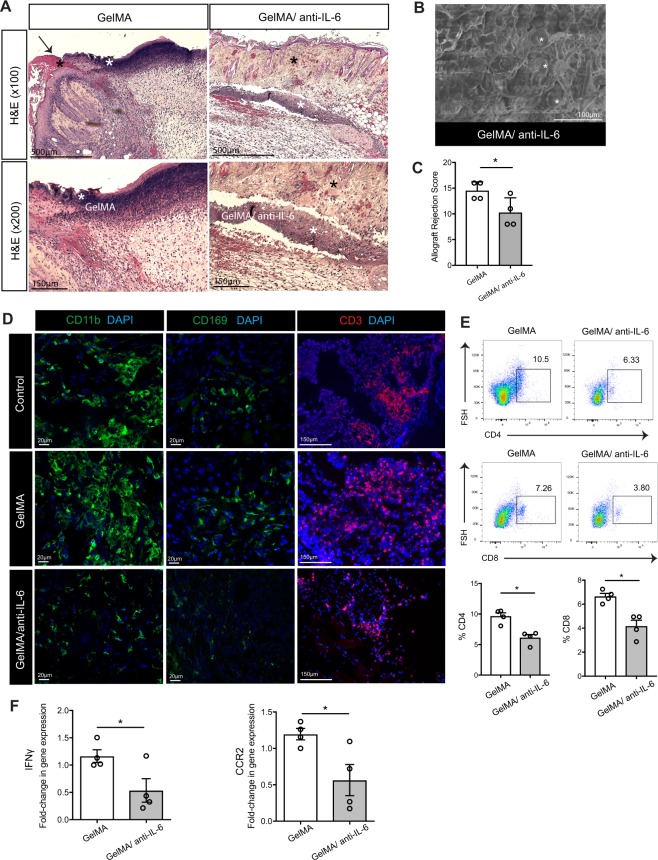
Co-transplantation with GelMA/anti-IL-6 IMB reduces T cell and monocyte infiltration into the allograft skin. The skin allografts were harvested at 7 days post-transplantation. (**A**) The skin allograft transplanted with GelMA demonstrated signs of rejection and necrosis, and it peeled off from recipient skin in the presence of massive cellular infiltration. However, the allograft skin transplanted with GelMA/anti-IL-6 attached to recipient skin with less pronounced cellular infiltration. (representative images from 4 different mice per group). (**B)** The SEM image of harvested allograft skin in GelMA/anti-IL-6 group showed maintenance of GelMA structure with the presence of red blood cells (white asterisk). (representative image from 2 different mice). (**C)** The skin allograft implanted with GelMA/anti-IL-6 had a significantly lower allograft rejection score compared to the one implanted with GelMA (GelMA versus GelMA/anti-IL-6, 14.5 ± 0.9 versus 10.2 ± 1.4, *p < 0.05, n = 4 mice/group). (**D)** Immunofluorescence staining of the skin allograft in control and GelMA group contained massive infiltrates of CD11b^+^, CD169^+^ and CD3^+^ cells in comparison to GelMA/anti-IL-6 group. (representative images from 4 different mice per group). (**E)** Flow cytometry analysis of the skin allografts revealed a higher percentage of CD4^+^ and CD8^+^ T cells in GelMA group compared to GelMA/anti-IL-6 group (GelMA versus GelMA/anti-IL-6, 9.6 ± 0.5 versus 6.1 ± 0.5 for CD4^+^, 6.6 ± 0.2 versus 4.1 ± 0.5 for CD8^+^, *p < 0.05, n = 4 mice/group). (**F)** Significantly lower gene expression of IFNγ and CCR2 were seen in the skin allograft harvested from GelMA/anti-IL-6 group compared to GelMA group (GelMA versus GelMA/anti-IL-6, 1.16 ± 0.1 versus 0.53 ± 0.2 for IFNγ, 1.20 ± 0.1 versus 0.56 ± 0.2 for CCR2, *p < 0.05, n = 4 mice/group).

The skin allograft transplanted with GelMA hydrogel demonstrated histological signs of rejection and necrosis, and it peeled off (black asterisk) from the skin of the recipient (Fig. 3A, left). The margin between the skins of the recipient and the allograft (black arrow) with the implanted GelMA hydrogel (white asterisk) contained massive cellular infiltration (Fig. 3A, left). In comparison, the cellular infiltration was less dense at the site of attachment between the skin allograft (black asterisk) transplanted with GelMA/anti-IL-6 IMB and the skin of the recipient (Fig. 3A, right). The magnified view of the implanted GelMA/anti-IL-6 IMB also showed lower migration of cells within the GelMA/anti-IL-6 hydrogel (white asterisk) (Fig. 3A, right). The scanning electron microscope (SEM) image of GelMA/anti-IL-6 harvested 7 days post-implantation from underneath the allograft skin showed maintenance of the GelMA structure, along with the presence of red blood cells (white asterisks) (Fig. 3B). The allograft rejection score, calculated as a combination of the severity of the vasculitis, folliculitis, dermal inflammation, and epidermal degeneration of the allograft, revealed less rejection of the skin allografts transplanted with GelMA/anti-IL-6 in comparison to those transplanted with GelMA (GelMA versus GelMA/anti-IL-6, 14.5 ± 0.9 versus 10.2 ± 1.4, *p < 0.05, n = 4 mice/group) (Fig. 3C).

Dendritic cells (DCs) and macrophages are known to play an initial and critical role in the induction of the alloimmune response through antigen presentation to naïve T cells. Immunofluorescent staining of the allograft skin transplanted with GelMA hydrogel as well as the control group revealed a similar massive infiltration by CD11b^+^ cells (Fig. 3D). Semi-quantitative measurement revealed that the GelMA group contained a larger area occupied by CD11b^+^ cells in comparison to the GelMA/anti-IL-6 group (Supplementary Figure 1A). CD169, known as sialoadhesin or sialic acid binding immunoglobulin-like lectin (Siglec) 1, is expressed strongly by specific macrophage subsets that exist primarily in the spleen and lymph nodes^49,50^. However, we observed many CD169^+^ macrophages in the skin allograft transplanted with GelMA hydrogel as well as the control group, in contrast to the few cells observed in the GelMA/anti-IL6 group (Fig. 3D) (Supplementary Figure 1B). Furthermore, the control and GelMA group contained more CD3^+^ cells, as compared to the GelMA/anti-IL-6 group (Fig. 3D). Flow cytometric analysis of the skin allograft harvested 7 days post-transplantation revealed a higher percentage of CD4^+^ and CD8^+^ T cells in the GelMA group, as compared to the GelMA/anti-IL-6 group, a difference that supported the histological findings (GelMA versus GelMA/anti-IL-6, 9.6 ± 0.5 versus 6.1 ± 0.5 for CD4^+^, 6.6 ± 0.2 versus 4.1 ± 0.5 for CD8^+^, *p < 0.05, n = 4 mice/group) (Fig. 3E). Furthermore, we examined the level of gene expression of inflammatory cytokines in the skin allograft. No significant difference in the expression of IL-17, CXCR3, CCR5 and CCL2 (data not shown) was found. However, we observed significantly lower levels of gene expression of IFNγ and CCR2 in the skin allograft harvested from the GelMA/anti-IL-6 group, compared to the GelMA group (GelMA versus GelMA/anti-IL-6, 1.16 ± 0.1 versus 0.53 ± 0.2 for IFNγ, 1.20 ± 0.1 versus 0.56 ± 0.2 for CCR2, *p < 0.05, n = 4 mice/group) (Fig. 3F).

### Local delivery of anti-IL-6 was associated with less severe inflammation in the DLN

**Figure 4:**
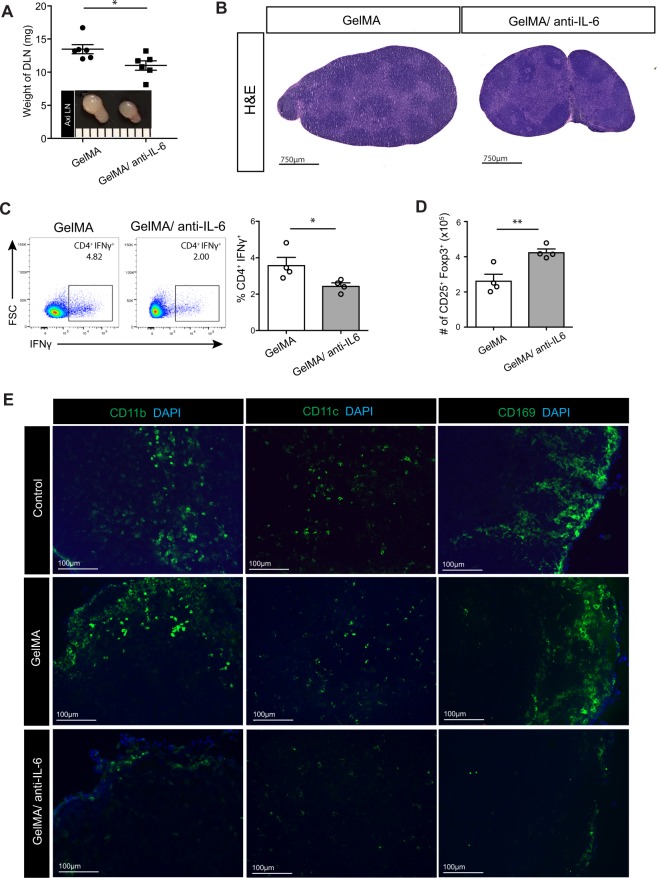
Local delivery of anti-IL-6 is associated with less severe inflammation in the DLN. The allograft skins were harvested at 7 days post-transplantation. (**A)** The DLNs harvested from GelMA/anti-IL-6 group were smaller and lighter than those from the GelMA group (GelMA versus GelMA/anti-IL-6, 13.5 ± 0.7 versus 11.0 ± 0.7, *p < 0.05, n = 6 LNs from 3 mice/group). (**B)** Light micrograph of H&E-stained DLN harvested from GelMA group revealed enlargement with massive cellular infiltration resulting in disorganization of B cell and T cell zones. However, the DLN harvested from GelMA/anti-IL-6 group was smaller in size with clearer margin between B cell and T cell zones. (representative images from 3 different mice per group). (**C)** Flow cytometric analysis revealed lower percentage of IFNγ-producing CD4^+^ T cells in GelMA/anti-IL-6 group in comparison to GelMA group (GelMA versus GelMA/anti-IL-6, 3.6 ± 0.4 versus 2.4 ± 0.2, *p < 0.05, n = 4 mice/group). (**D)** Flow cytometric analysis revealed a higher number of Treg cells (CD25^+^Foxp3^+^ cells) in DLNs harvested from GelMA/anti-IL-6 group than those from GelMA group (GelMA versus GelMA/anti-IL-6, 2.6 × 10^5^ ± 0.3 × 10^5^ versus 4.2 × 10^5^ ± 0.2 × 10^5^, **p < 0.01, n = 4 mice/group). (**E)** Fluorescent micrographs of DLNs revealed more CD11b^+^, CD11c^+^ and CD169^+^ cells in the subcapsular sinus of control and GelMA group in comparison to GelMA/anti-IL-6 group. (representative images from 4 different mice per group).

Next, we examined how implantation of GelMA/anti-IL-6 IMB underneath the skin allograft affects draining lymph nodes (DLNs). The ipsilateral axillary lymph node (Axi LN) and brachial lymph node on the side of skin transplanted side were designated as the DLNs. As shown in Fig. 4A, the DLNs harvested from the GelMA/anti-IL-6 group were smaller than those in the GelMA group. The DLNs of the GelMA/anti-IL-6 group were also lighter in comparison to the GelMA group (GelMA versus GelMA/anti-IL-6, 13.5 ± 0.7 mg versus 11.0 ± 0.7 mg, *p < 0.05, n = 6 LNs from 3 mice/group) (Fig. 4A). The DLNs harvested from the GelMA group were enlarged and showed characteristics of activation (Fig. 4B, left). However, the DLNs harvested from the GelMA/anti-IL-6 group were smaller in size and contained a clearer margin between the B cell and T cell zones (Fig. 4B, right). Flow cytometric analysis of the DLN revealed no difference in the populations of CD4^+^ and CD8^+^ T cells between the groups (GelMA versus GelMA/anti-IL-6, 1943 ± 0.9 versus 19.3 ± 0.3, *p* = ns for CD4^+^, 19.7 ± 1.8 versus 19.0 ± 0.5, *p* = ns for CD8^+^, respectively, n = 4 mice/group) (Supplementary Figure 2A). However, flow cytometric analysis revealed a higher percentage of IFNγ-producing CD4^+^ T cells in the DLNs of the GelMA/anti-IL-6 group, as compared to those of the GelMA group (GelMA versus GelMA/anti-IL-6, 3.6 ± 0.4 versus 2.4 ± 0.2, *p < 0.05, n = 4 mice/group) (Fig. 4C). Interestingly, we noticed that the DLNs from GelMA/anti-IL-6 group contained a higher number of Treg cells (CD25^+^Foxp3^+^ cells) in comparison to those from the GelMA group (GelMA versus GelMA/anti-IL-6, 2.6 × 10^5^ ± 0.3 × 10^5^ versus 4.2 × 10^5^ ± 0.2 × 10^5^, **p < 0.01, n = 4 mice/group) (Fig. 4D).

Immunofluorescence staining of the DLN revealed many more CD11b^+^, CD11c^+^ and CD169^+^ cells in the subcapsular sinus in the control and GelMA groups, as compared to the GelMA/anti-IL-6 group (Fig. 4E). Next, we examined the expression of the chemokines CCL2 and CXCL9 in the DLNs. The expression of these chemokines in the DLNs from the GelMA/anti-IL-6 group was lower in comparison to the GelMA group, but the differences did not attain statistical significance (Supplementary Figure 2B).

### Local release of anti-IL-6 from IMB suppressed LN matrix accumulation

**Figure 5:**
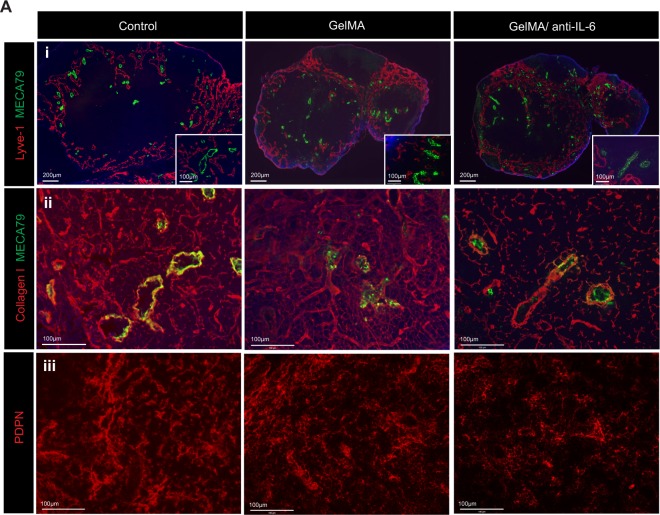
Local release of anti-IL-6 from IMB suppresses LN fibrosis. The skin allografts were harvested at 7 days post-transplantation. (**A-i**) Lymphatic vessel expansion (Lyve-1^+^) and HEV elongation (MECA79^+^) were similar between all groups. (**A-ii,iii**) Dense staining of collagen I and PDPN was seen in DLNs harvested from control and GelMA group compared to those from GelMA/anti-IL-6 group. (representative images from 4 different mice per group).

Continuous inflammation has been known to induce matrix accumulation in the LN^51^. We observed similar expansion of Lyve-1^+^ lymphatic vessels and elongation of peripheral node addressin (PNAd)^+^ high endothelium venules (HEVs), stained with the MECA79 antibody, in the DLNs from control, GelMA and GelMA/anti-IL-6 groups (Fig. 5A-i). Next, we stained the DLNs for extra cellular matrix proteins, such as collagen I and podoplanin (PDPN). Areas of collagen I^+^ (Fig. 5A-ii) and PDPN^+^ (Fig. 5A-iii) staining were denser and thicker in the DLNs harvested from both the control and GelMA groups, as compared to those from the GelMA/anti-IL-6 group.

### Local release of anti-IL-6 did not affect the systemic immune response

Finally, we examined the systemic impact of the local release of anti-IL-6 by examining the spleens harvested 7 days post-transplantation. No difference in the percentages of CD4^+^ and CD8^+^ T cells in the spleens between the two groups was observed (GelMA versus GelMA/anti-IL-6, 20.9 ± 0.9 versus 20.9 ± 0.5, *p* = ns for CD4, 15.5 ± 0.7 versus 17.3 ± 0.8, *p* = ns for CD8, respectively) (Supplementary Figure 3A). Also, no difference in the expression level of CD69 by either CD4^+^ or CD8^+^ T cells was found between the groups (GelMA versus GelMA/anti-IL-6, 17.0 ± 0.5 versus 16.2 ± 0.7, *p* = ns for CD4^+^CD69^+^, 14.5 ± 0.6 versus 15.6 ± 0.9, *p* = ns for CD8^+^CD69^+^, respectively) (Supplementary Figure 3B and C).

We examined further the differences between IFNγ-producing CD4^+^ T cells and CD4^+^CD25^+^Foxp3^+^ (Treg) cells in the spleens from the two groups. However, no difference was found in either cell type between the groups (GelMA versus GelMA/anti-IL-6, 0.6 ± 0.06 versus 0.5 ± 0.05, *p* = ns for CD4^+^IFNγ^+^, 5.3 ± 0.9 versus 4.6 ± 0.4, *p* = ns for CD4^+^CD25^+^Foxp3^+^, respectively) (Supplementary Figure 3D and E).

## Discussion

While skin regenerative therapies have improved markedly, trauma patients suffering from severe burns still experience high mortality^52^. A significant effort has been made to mimic the physical characteristics of skin, but synthetic and semi-biological skin substitutes still do not replicate skin with complete biological characteristics. Indeed, in the absence of a skin autograft, the only skin tissue that is a truly full biological substitute is a skin allograft. In addition to its role as a protective barrier against infectious agents, the skin contains a sophisticated innate immune system, which interacts together with circulatory immune cells to sense the presence of exogenous insults to the body and can trigger rejection of a skin allograft^53^. Despite a possible delay in skin allograft rejection due to the immunocompromised state of burn patients, most of these allografts still reject within 7 days to 2 weeks. While some attempts have been made to use immunosuppressive medications to increase the life span of allografts for a successful transition to autografts, the use of immunosuppression increases the rate of infection markedly; and following discontinuation of immunosuppression, these grafts are ultimately rejected^6,54^.

An under-explored idea that may result potentially in substantial improvement of transplantation outcomes is the design of novel strategies to reduce intra-graft inflammation^14^. Amongst the various inflammatory cytokines, IL-6 is one of the most pleotropic cytokines known^55,56^. IL-6 plays a critical role in the pathogenesis of allograft rejection in general, and targeting IL-6 results in reduced acute rejection and amelioration of chronic vascular injuries^56–63^. Targeting IL-6 also promotes the tolerogenic effect of co-stimulatory blockade^60,64^. Mechanistically, IL-6 acts in concert with inflammatory cytokines to induce differentiation of CD4^+^ cells towards pathogenic T cells^65^.

We generated an adhesive immunomodulatory biomaterial (IMB) that elutes anti-IL-6 gradually underneath the skin allograft. The local release of anti-IL-6 at the site of wound can reduce inflammation effectively around the skin allograft implanted with the hydrogel. Our data showed that incorporation of anti-IL-6 to GelMA hydrogels has no significant effects on the mechanical properties of the hydrogel. This finding is consistent with the results of previous studies, in which the addition of small molecules (e.g. growth factors, cytokines, peptides) had negligible effects on the mechanical properties of the hydrogel^38,66,67^. Future studies are needed to assess further the impact of biomaterials that have stronger mechanical support and can simultaneously provide an optimal immunomodulatory environment.

The skin allografts transplanted with GelMA/anti-IL-6 IMB survived almost twice as long as the control group. Remarkably, these allografts had a much more favorable outcome than the skin allografts in mice that were treated systemically with an 8-fold higher dosage of anti-IL-6. This finding highlights the effectiveness and significance of local release of anti-inflammatory agents in the prevention of allograft rejection, and it demonstrates consistency with several previous reports that indicated the direct association between intra-graft inflammation and graft rejection^14,68,69^. Histologic observation of the allograft skin revealed fewer CD11b^+^ cells, CD169^+^ cells, and CD3^+^ cells in the GelMA/anti-IL-6 group, as compared to the control and GelMA group. DCs and macrophages are initial and critical responders against allo-antigen. Especially in the skin, Langerhans cells (LCs) are highly specialized in antigen uptake and act as skin-resident antigen-presenting cells (APCs)^70,71^. CD169^+^ macrophages are known to reside in the subcapsular sinus and marginal sinus of secondary lymphoid organs in general, and these are necessary for pathogen removal^72^. A recent report indicated that CD169^+^ macrophages collaborate with DCs to activate CD8^+^ T cells and transfer antigens between each other^73^. The pro-inflammatory cytokine IL-6 plays a main role in the activity of these DCs and macrophages. IL-6 polarizes macrophages into the M1 subtype, which results in subsequent production of IL-6 by these macrophages and activation of lymphocytes.

We also observed smaller DLNs with fewer CD11b^+^, CD11c^+^ and CD169^+^ cells in the GelMA/anti-IL-6 group. Furthermore, the DLNs from the GelMA/anti-IL-6 group contained significantly fewer IFNγ-producing CD4^+^ T cells, but more Treg cells. IL-6 has been shown to suppress Treg induction^74^. Activation of LNs in the control and GelMA groups was noted by an excessive accumulation of matrix materials, as compared to the GelMA/anti-IL-6 group. Besides the sustained release of anti-IL-6, GelMA also provides a microenvironment for the integration of reparative cells and new vascularization that facilitates the adoption of new skin^40,75^. Nanoparticles, despite providing slow release, may not support the wound and allografts in the multifaceted fashion of biomaterials such as GelMA. However, combining these two strategies may increase their efficacy in a synergistic manner.

Given the robust nature of skin transplant models, strategies that show promising results in prolonging skin graft survival in mice typically carry high significance in humans. It is likely that the prolongation of skin graft survival that we observed in our mouse model would be enhanced further in burn patients who are inherently immunosuppressed. An alternative approach is to incorporate other immunomodulatory factors (i.e., CTLA4-Ig) or drugs (tacrolimus and/or sirolimus) into the hydrogel. We postulate that extending the survival of skin allografts will allow the patients to become more stable to allow them to receive autografts (and/or autologous cultured skin). There is possibility that such patients may indefinitely accept the implanted skin allograft without or with low dose immunosuppression. Several burn patients have accepted skin allografts indefinitely, sometimes in the absence of immunosuppressive agents; others have remained tolerant following discontinuation of their immunosuppressive regimen^54,76–80^. Thus, maximizing the life span of skin allografts may increase the likelihood of tolerance and not require the use of any systemic immunosuppressive agents. Here, we conclude that local immunomodulatory delivery of anti-IL-6 could serve as a basis for future localized delivery of immune therapeutics to prolong skin allograft survival.

## Materials and Methods

### Materials

Gelatin (Type A, bloom 300 from porcine skin), methacrylic anhydride (MA, 94%), triethanolamine (TEA, 98%), N-vinylcaprolactam (VC, 98%), and Eosin Y (2′,4′,5′,7′-tetrabromofluorescein) were purchased from Sigma-Aldrich (USA) and used as received. Anti-mouse IL-6 receptor antibody (referred to as anti-IL-6, cMR16-1; courtesy of Genentech) was used for either GelMA/anti-IL-6 synthesis or systemic *in vivo* treatment (100 μg/mouse, intraperitoneal injection on day 0–3 and then on alternate days until day 11).

### Gelatin methacryloyl (GelMA) synthesis

Type A porcine skin gelatin was dissolved in Dulbecco’s phosphate buffered saline (DPBS, Gibco^®^) at 60 °C, yielding a 5% (w/v) solution. MA (8 mL) was added to the gelatin solution dropwise while stirring at 50 °C, which was allowed to react for 2 h. To stop the reaction, the solution was diluted by adding warm (50 °C) DPBS, followed by dialysis against distilled water using dialysis tubing (MWCO ~ 12–14 kDa cutoff, Spectrum^®^ Laboratories) for 1 week at 50 °C for purification. The solution was lyophilized for 5 days, yielding white foam.

### GelMA/anti-IL-6 immunomodulatory biomaterial preparation

Lyophilized GelMA was dissolved in DPBS containing TEA, VC, and anti-IL-6, and separately, Eosin Y disodium salt was dissolved in DPBS. The GelMA/TEA/VC/anti-IL-6 prepolymer solution was mixed with the Eosin Y solution, yielding a hydrogel solution containing GelMA (5% w/v), TEA (1.5% w/v), VC (1% w/v), Eosin Y (0.1 mM), and 100 μg of anti-IL-6 (0.4% w/v). Hydrogels were formed through visible light (450–550 nm)-mediated photocrosslinking of pre-gel solutions for 4 minutes using a LS1000 FocalSeal xenon light source (100 mW cm^−2^, Genzyme).

### Characterization of hydrogel mechanics

Mechanical testing for GelMA and GelMA/anti-IL-6 IMB was performed using an Instron 5943 mechanical tester. Both the elastic modulus and compressive modulus were analyzed (n ≥ 3). Hydrogels were formed by photocrosslinking 70 μL of the pre-gel solutions in polydimethylsiloxane (PDMS) molds for 4 min. Rectangular molds (length: 12.00 mm, width: 5.00 mm, depth: 1.25 mm) were used for tensile testing, while cylindrical ones (diameter: 6.00 mm, height: 3.00 mm) were used for compression testing. For compression testing, hydrogels were placed between two compression plates and compressed at a rate of 1 mm/min. Compression strain and compressive stress were registered during each test, using Bluehill software. Compression modulus was calculated from the slope of the initial linear region in the stress-strain curve. For tensile testing, hydrogels were secured between two tensile grips and stretched at a rate of 1 mm/min until failure. The elastic modulus was then calculated from the initial linear region of the stress-strain curve, taken from the 0.4–0.6 mm^−1^ of the strain.

### Anti-IL-6 release study

From the GelMA/TEA/VC/Anti-IL6/Eosin Y solution, 25 μL was placed into cylindrical PDMS molds (diameter: 6.00 mm; height: 2.50 mm), followed by photocrosslinking using visible light exposure for 4 min. The hydrogel was then immersed and maintained in 2 mL of DPBS at 37 °C in an incubator. At each time point, 100 μL of supernatant was collected using a pipette and examined using an ELISA kit (IgG Mouse ELISA Kit, Abcam) according to the manufacturer’s method to quantify the release of anti-IL-6. The supernatants were diluted so that the anti-IL-6 concentration fell within the range of the ELISA.

### Mice

All animal experiments and methods were performed in accordance with the relevant guidelines and regulations approved by the Institutional Animal Care and Use Committee of Brigham and Women’s Hospital, Harvard Medical School, Boston, MA (protocol number: 2016N000167/04977). Female C57BL/6 and BALB/c mice were purchased from Jackson Laboratory (Bar Harbor, ME, USA) and used at 7–9 weeks of age.

### Murine skin transplantation

A full-thickness trunk skin graft was harvested from a BALB/c donor mouse. 1 cm^2^ of left upper back skin was removed from the recipient C57BL/6 mouse. For both the GelMA and GelMA/anti-IL-6 groups, 25 μl of the corresponding solution was poured over the wound of the recipient C57BL/6 mouse and crosslinked through visible light exposure for 4 minutes. Harvested BALB/c donor skin was trimmed, placed onto the cross-linked GelMA and sutured. Transplanted skin was secured with a bandage for 7 days.

### Flow cytometry

Flow cytometric analysis of skin allograft, draining lymph node (DLN) and spleen was performed, and each leukocyte population was quantified. CD4 (RM4-5, #100559, 1:400), CD8 (53-6.7, #100734, 1:400), CD69, CD25 (PC61.5, #12-0251-81B, 1:400), Foxp3 (FJK-16s, #11-5773-82, 1:300) and Annexin-PE (51-65875X) were purchased from BD Biosciences, San Jose, CA. For intracellular cytokine staining, cells were stimulated *ex vivo* with phorbol 12-myristate 13-acetase (PMA, 50 ng/ml) and ionomycin (500 ng/ml) in combination with GolgiStop for 4 hours, then permeabilized and stained with fluorochrome conjugated antibodies against IFNγ. Cells were run on FACSCanto II (BD Biosciences, Franklin Lakes, NJ). Data were analyzed using FlowJo software.

### Immunohistochemistry

Skin grafts and DLNs harvested at designated time points post-skin transplantation were fixed in formalin and embedded to paraffin block. Samples were cut into 5 μm-thick sections and stained with hematoxylin and eosin (H&E). For the skin allograft rejection score, an combination of the severity of the vasculitis (grade 0: normal, grade 1: mild, grade 2: moderate, grade 3: severe, grade 4: massive, necrosis or hemorrhage), folliculitis (grade 0–4), dermal inflammation (grade 0–4) and epidermal degeneration (grade 0–4) was calculated and plotted to the graph (Total from 0–16).

### Immunofluorescent staining

Skin grafts and DLNs harvested at designated time points post-skin transplantation were preserved in Optimal Cutting Temperature compound and stored at −80 °C. Samples were cut into 5 μm-thick sections and stained with Lyve-1 (Abcam, #ab14917; 1:300), CD11b (Abcam, #ab8878; 1:250), CD11c (Biolegend, #101206; 1:100), CD169 (Biolegend, #142406; 1:100), CD3 (Abcam, #ab16669; 1:250), MECA79 (Novus Biologicals, #NB100-77673; 1:200), Collagen I (Novus Biologicals, #NB600-408; 1:200), and PDPN (R&D System, #AF3244; 1:200) antibodies. For the quantification of images, all images were automatically processed using ImageJ (NIH) and split into RGB channels. Auto threshold was used to convert intensity values of the immunefluorescent stain into numeric data.

### Real-time PCR experiments

The skin allograft or DLNs were harvested and immediately snap frozen in liquid nitrogen. RNA was extracted with TRIZOL (Invitrogen), and first strand cDNA was synthesized using 1 μg of RNA and High-Capacity Reverse Transcriptase (Invitrogen). RT-PCR was performed with SYBR Green PCR reagents on a Biorad detection system. RNA levels were normalized to the level of GAPDH and calculated as delta-delta threshold cycle (ΔΔCT). Primers used for RT-PCR are listed as bellows; GAPDH-For:AGCCACATCGCTCAGACAC, GAPDH-Rev:GCCCAATACGACCAAATCC, IFNγ-For:TTGAGGTCAACAACCCACAG, IFNγ-Rev:TCAGCAGCGACTCCTTTTC, CCR2-For:ACACCCTGTTTCGCTGTAGG, CCR2-Rev:GATTCCTGGAAGGTGGTCAA, CCL2-For:GAAGGAATGGGTCCAGACAT, CCL2-Rev:ACGGGTCAACTTCACATTCA, CXCL9-For:CCGAGGCACGATCCACTAC, CXCL9-Rev:AGGCAGGTTTGATCTCCGTT.

### Statistics

Kaplan-Meier survival graphs were constructed, and log rank comparison of the groups was used to calculate *p*-values for survival comparisons between the various groups. Data analysis was performed using GraphPad Prism (GraphPad Software, Inc., San Diego, CA). Differences between groups were evaluated by student’s *t*-test to determine significance. *p < 0.05 was considered a significant difference.

## Supplementary information

Supplementary Figure 1-3

## Data Availability

All data generated or analyzed during this study are available from the corresponding author on reasonable request.
