# Supplementary material for: Anti-IL-6 eluting immunomodulatory biomaterials prolong skin allograft survival
Source: Sci Rep. 2019 Apr 25;9:6535. doi: 10.1038/s41598-019-42349-w (PMC6484015; doi:10.1038/s41598-019-42349-w)
Supplement: Supplementary file 1 — Supplementary Figure 1-3 [file 41598_2019_42349_MOESM1_ESM.pdf]

Anti-IL-6 eluting immunomodulatory biomaterials prolong skin allograft survival

Mayuko Uehara[1‡], Xiaofei Li[1‡], Amir Sheikhi[2,3,4,5], Nooshin Zandi[2,3,6], Brian Walker[4,7], Bahram Saleh[7], Naima Banouni[1], Farideh Ordikhani[1], Li Dai[1], Merve Yonar[1], Ishaan Vohra[1], Vivek Kasinath[1], Dennis P. Orgill[8], Ali Khademhosseini[2,3,4,5,9,10,11,12], Nasim Annabi[2,3,5,7,9], Reza Abdi[1]*

[1]Transplantation Research Center, Renal Division, Brigham and Women's Hospital, Harvard Medical School, Boston, MA 02115, USA.

[2]Biomaterials Innovation Research Center, Division of Biomedical Engineering, Department of Medicine, Brigham and Women's Hospital, Harvard Medical School, Cambridge, MA 02139, USA.

[3]Harvard-MIT Division of Health Sciences and Technology, Massachusetts Institute of Technology, Cambridge, MA 02139, USA.

[4]Department of Bioengineering, University of California, Los Angeles, CA 90095, USA.

[5]Center for Minimally Invasive Therapeutics (C-MIT), California NanoSystems Institute (CNSI), University of California, Los Angeles, CA 90095, USA.

[6]Institute for Nanoscience and Nanotechnology, Sharif University of Technology, Tehran, Iran.

[7]Department of Chemical Engineering, Northeastern University, Boston, MA 02115, USA.

[8]Department of Plastic Surgery, Brigham and Women's Hospital, Harvard Medical School, Boston, MA 02115, USA.

[9]Department of Chemical and Biomolecular Engineering, University of California Los Angeles, Los Angeles, California 90095, USA.

[10]Department of Radiological Sciences, David Geffen School of Medicine, University of California, Los Angeles, CA 90095, USA.

[11]Department of Bioindustrial Technologies, College of Animal Bioscience and Technology, Konkuk University, Seoul, 143-701, Republic of Korea.

[12]Center of Nanotechnology, Department of physics, King Abdulaziz University, Jeddah, 21569, Saudi Arabia.

‡ These authors contributed equally to this work.

*Address correspondence to:

Reza Abdi, MD

Transplantation Research Center, Brigham and Women's Hospital

221 Longwood Ave, Boston MA 02115, USA

Tel: 617-732-5259, Fax: 617-732-5254, E-mail: rabdi@rics.bwh.harvard.edu

Supplementary Figure Legend

Supplementary Figure 1) Semi-quantitative measurement of $CD11b^+$ and $CD169^+$ cells in the skin allografts

A) Region of $CD11b^+$ staining was significantly larger in skin allografts from GelMA group in comparison to GelMA/anti-IL-6 group (GelMA versus GelMA/anti-IL-6, 56.7±6.6 versus 36.6±3.4, *$p<0.05$, n=10 images from 4 mice/group). **B)** $CD169^+$ region was significantly larger in skin allografts of GelMA group compared to GelMA/anti-IL-6 (GelMA versus GelMA/anti-IL-6, 35.0±7.4 versus 17.0±3.6, *$p<0.05$, n=10 images from 4 mice/group).

Supplementary Figure 2) Flow cytometric and gene expression analysis of DLN

A) Flow cytometric analysis revealed no significant difference in the population of $CD4^+$ and $CD8^+$ T cells in the DLNs between the two groups (GelMA versus GelMA/anti-IL-6, 1943±0.91 versus 19.3±0.25, $p=0.98$ for $CD4^+$, 19.7±1.8 versus 19.0±0.5, $p=0.74$ for $CD8^+$, respectively, n=4 mice/group) in DLN. **B)** The expression of the chemokines CCL2 and CXCL9 in DLN from GelMA/anti-IL-6 group was significantly lower in comparison to GelMA group (GelMA versus GelMA/anti-IL-6, 1.1±0.1 versus 0.8±0.1, $p=0.15$ for CCL2, 1.3±0.1 versus 0.9±0.1, $p=0.09$ for CXCL9, respectively, n=3 mice/group).

Supplementary Figure 3) Flow cytometric analysis of spleen

A) Flow cytometric analysis revealed no significant difference in the percentages of $CD4^+$ and $CD8^+$ T cells in the spleens between the two groups (GelMA versus GelMA/anti-IL-6, 20.9±0.9 versus 20.9±0.5, $p=0.99$ for CD4, 15.5±0.7 versus 17.3±0.8, $p=0.15$ for CD8, respectively, n=4 mice/group). **B-C)** The expression in the spleen of $CD69^+$ by either $CD4^+$ and $CD8^+$ T cells also

was not significantly different between the groups (GelMA versus GelMA/anti-IL-6, 17.0±0.5 versus 16.2±0.7, p=0.36 for $CD4^+CD69^+$, 14.5±0.6 versus 15.6±0.9, p=0.33 for $CD8^+CD69^+$, respectively, n=4 mice/group). **D-E)** Flow cytometric analysis revealed no significant differences in the IFNγ-producing $CD4^+$ T cells and $CD4^+CD25^+Foxp3^+$ (Treg) cells in the spleens between the two groups (GelMA versus GelMA/anti-IL-6, 0.6±0.06 versus 0.5±0.05, p=0.41 for $CD4^+IFN\gamma^+$, 5.3±0.9 versus 4.6±0.4, p=0.56 for $CD4^+CD25^+Foxp3^+$, respectively, n=4 mice/group).

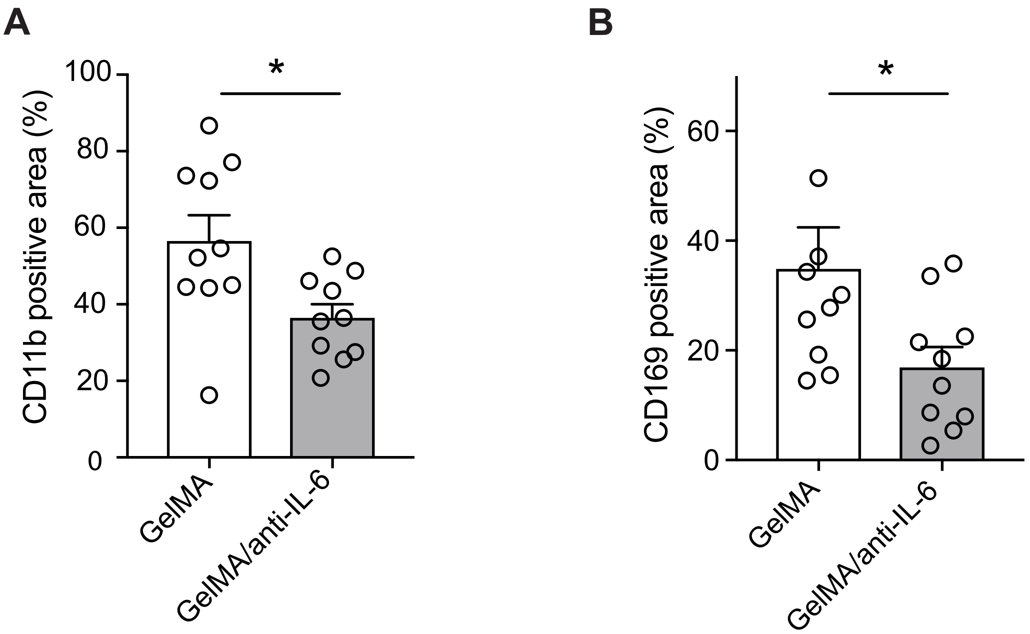

Supplementary Figure 1

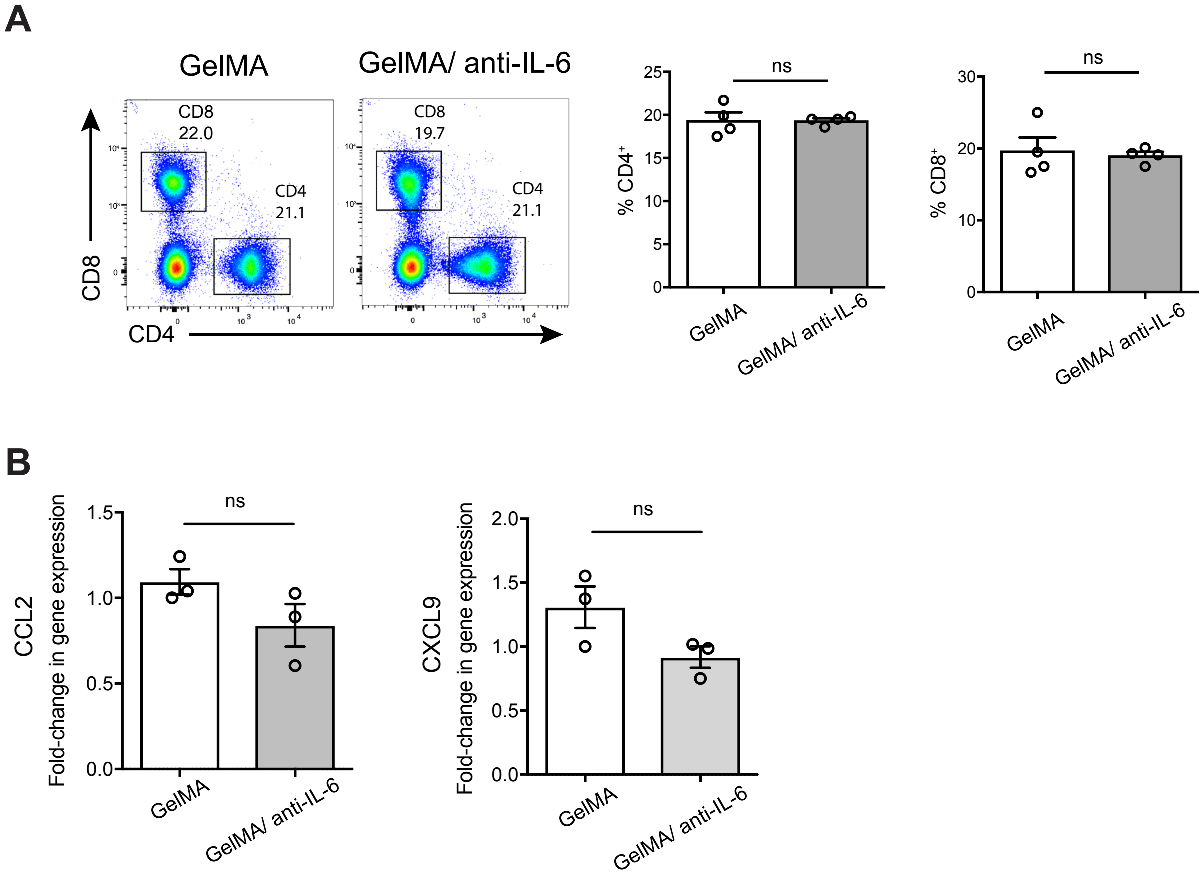

Supplementary Figure 2

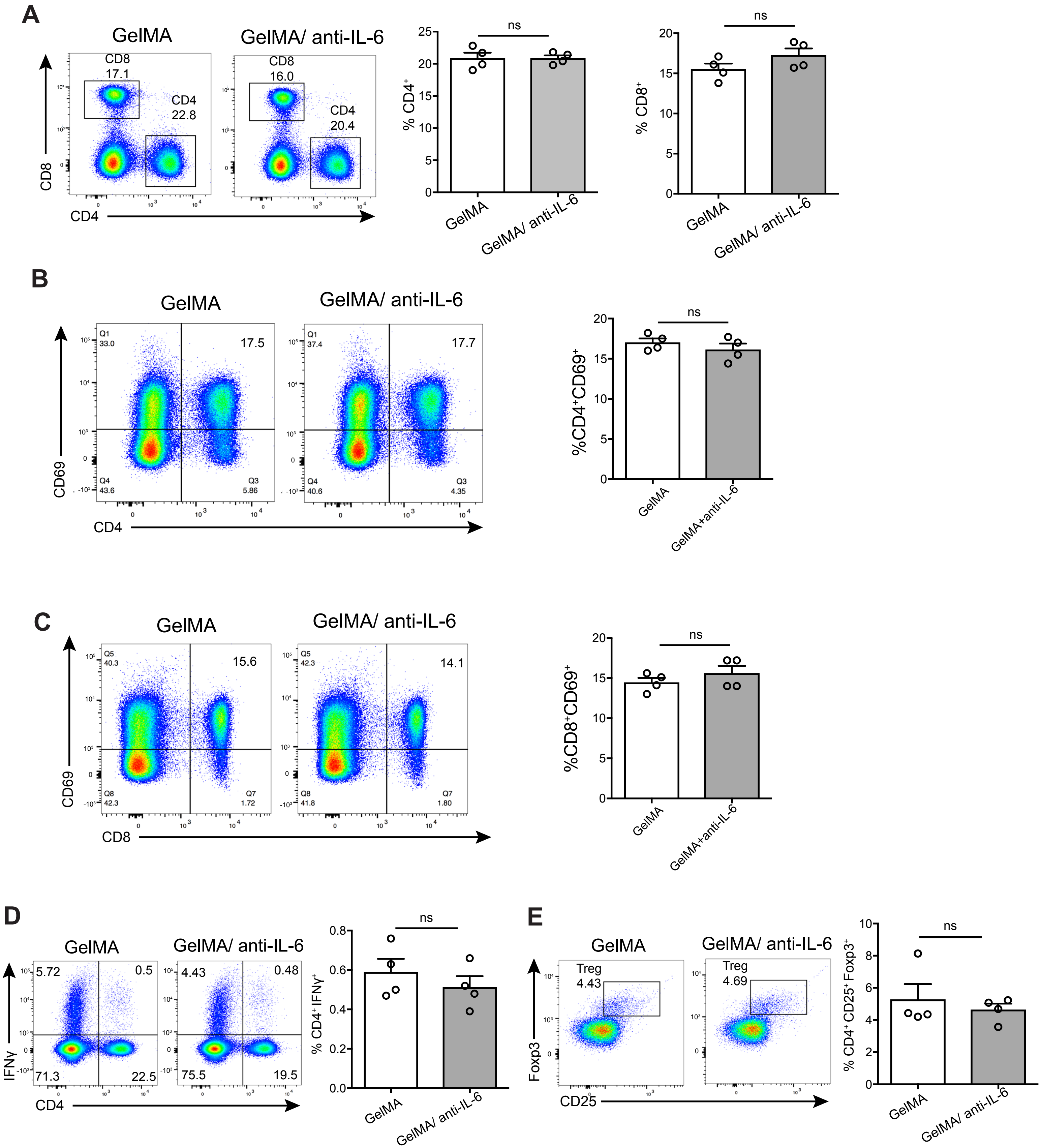

Supplementary Figure 3
